# Bildgebung des Retinoblastoms: Aktueller Stand der Technik und Ausblick in die Zukunft

**DOI:** 10.1007/s00117-022-01052-0

**Published:** 2022-08-15

**Authors:** Bernd Schweiger, Sophia Göricke, Petra Ketteler, Eva Biewald, Raimund Kottke, Selma Sirin

**Affiliations:** 1 Institut für Diagnostische und Interventionelle Radiologie und Neuroradiologie, Universitätsklinikum Essen, Essen, Deutschland; 2 Klinik für Pädiatrische Hämatologie und Onkologie, Universitätsklinikum Essen, Essen, Deutschland; 3 Klinik für Augenheilkunde, Universitätsklinikum Essen, Essen, Deutschland; 4 Abteilung für Bilddiagnostik, Universitäts-Kinderspital Zürich, Zürich, Schweiz

**Keywords:** Augentumor, Kinder, Malignom, Hochaufgelöste Magnetresonanztomographie, Tumorprädispositionssyndrom, Eye neoplasms, Children, Malignant neoplasms, High-resolution magnetic resonance imaging, Tumor predisposition syndrome

## Abstract

**Hintergrund:**

Das Retinoblastom ist der häufigste bösartige Augentumor im Kindesalter und in bis zu 40 % der Fälle mit einem Tumorprädispositionssyndrom assoziiert (*RB1*-Mutation). Die Bildgebung ist ein wichtiger Bestandteil der diagnostischen Evaluation von Kindern mit Retinoblastom zum Zeitpunkt der Diagnose und im Follow-up.

**Ziel der Arbeit:**

Diese Übersichtsarbeit soll den aktuellen Stand der Technik und wichtige diagnostische Aspekte der radiologischen Bildgebung von Kindern mit Retinoblastom aufzeigen mit einem kurzen Ausblick in die Zukunft. Zusätzlich wird ein Überblick über die allgemeine klinische Diagnostik und die Therapiemöglichkeiten gegeben.

**Material und Methoden:**

Basis der Arbeit ist die Recherche in verschiedenen Literaturdatenbanken sowie eigene Erfahrungen in der Bildgebung des Retinoblastoms.

**Schlussfolgerung:**

Hochaufgelöste MRT-Bildgebung ist die Bildgebungsmodalität der Wahl bei Kindern mit Retinoblastomen zum Zeitpunkt der Diagnose (Abklärung der Diagnose/möglicher Differenzialdiagnosen, Evaluation der Tumorausdehnung okulär und intrakraniell) und im Follow-up. CT-Untersuchungen sind trotz der charakteristischen Verkalkungen zur Diagnostik nicht mehr indiziert. Da Retinoblastome bis zu 40 % mit Tumorprädispositionssyndromen assoziiert sind, sollte stets auch eine genetische Abklärung erfolgen.

## Abstract (en)

**Background:**

Retinoblastoma is the most common malignant eye tumor in children and is associated with tumor predisposition syndrome (*RB1* mutation) in up to 40% of cases. Imaging is an important part of the diagnostic workup of children with retinoblastoma both during the initial diagnosis and follow-up.

**Objectives:**

The goal of this review is to present the current state-of-the-art regarding imaging of children with retinoblastoma, including technical background and diagnostic clues with a brief discussion of future prospects. In addition, we summarize the general clinical diagnostic workup and therapeutic options.

**Materials and methods:**

Review of the literature and our own experience in the imaging of retinoblastoma.

**Conclusion:**

High-resolution magnetic resonance imaging (MRI) is the imaging modality of choice in children with retinoblastoma for diagnosis (estimation of diagnosis/differential diagnosis, evaluation of local and intracranial tumor extension) and during follow-up. Despite the characteristic calcifications, computed tomography (CT) examinations are no longer indicated in these patients. Due to the high association with tumor predisposition syndrome, genetic counselling is recommended.

Bösartige Augentumoren mit dem Hauptvertreter Retinoblastom machen nur 2 % der Malignome im Kindesalter aus, sind jedoch in bis zu 40 % der Fälle mit einem Tumorprädispositionssyndrom (TPS) assoziiert. Da diese Kinder neben dem Retinoblastom ein höheres Risiko für weitere Malignome tragen, sollten sie erfasst, die Familien genetisch beraten und im Verlauf ihres Lebens begleitet werden.

Die hochaufgelöste Magnetresonanztomographie (MRT) ist die Bildgebungsmodalität der Wahl beim Retinoblastom. In diesem Beitrag wird ein Überblick über Diagnose und Therapie gegeben, mit Fokus auf den aktuellen Stand der Bildgebung und einem Ausblick in die Zukunft.

## Das Retinoblastom

Das Retinoblastom ist der häufigste bösartige Tumor des Auges im Kindesalter mit etwa 7000–8000 neuen Fällen pro Jahr weltweit und etwa 40 neuen Fällen in Deutschland [1, 2]. Das Retinoblastom tritt in 2 Formen auf: unilateral (ein Auge betreffend) und bilateral. Der Hauptteil der Patienten erkrankt im frühen Kindesalter (medianes Erkrankungsalter bei unilateraler Form: 27 Monate, bei bilateraler Form: 15 Monate); nach dem 5. Lebensjahr tritt die Erkrankung selten auf [2].

Die Prognose des Retinoblastoms hat sich in den westlichen Ländern in den letzten Jahrzehnten durch die stetige Weiterentwicklung der diagnostischen und therapeutischen Möglichkeiten sowie der Früherkennung deutlich gebessert, aktuell beträgt die 15-Jahres-Überlebensrate in Deutschland 98 %. In Afrika hingegen werden weiter Mortalitätsraten von 70 % (1254 Todesfälle pro Jahr), in Asien 39 % (1591 Todesfälle pro Jahr) genannt [3]. Auch wenn sie überleben, haben Kinder mit der Diagnose eines Retinoblastoms mit Spätfolgen zu kämpfen, insbesondere mit Visusverlust/Visusminderung, Gesichtsdeformitäten, posttherapeutischen Folgen wie Ototoxizität, Zweitmalignomen sowie (neuro)psychologischen Problemen, wodurch es zu einer relevanten Beeinträchtigung der Lebensqualität kommen kann.

Da es sich bei etwa 40 % der Fälle um hereditäre Retinoblastome handelt, spielt – insbesondere bei bilateraler Erkrankung – die Genetik eine große Rolle [4]. Retinoblastome entstehen durch ein abnormes und unkontrolliertes Wachstum von Retinoblasten, retinalen Vorläuferzellen, aufgrund einer Mutation im *RB1*-Tumorsuppressions-Gen auf Chromosom 13. Bei nichthereditären Retinoblastomen sind somit zwei unabhängige somatische *RB1*-Mutationen zur Tumorentstehung erforderlich, während beim hereditären Retinoblastom aufgrund des bereits vorhandenen Verlustes eines der *RB1*-Allele eine Mutation in dem anderen *RB1*-Allel ausreicht. Kinder mit hereditärer Erkrankung haben ein hohes Risiko für multiple bilaterale Retinoblastome, die Entwicklung eines trilateralen Retinoblastoms (s. unten) sowie ein erhöhtes Risiko für extraokuläre Tumorerkrankungen im weiteren Leben, insbesondere für Weichteilmalignome und Osteosarkome. Die kumulative Inzidenz für Zweitmalignome beträgt bis zu 50 % bei Kindern mit bilateralen Retinoblastomen und positiver Familienanamnese 50 Jahre nach der Erstdiagnose [5].

## Diagnostik

Die Abklärung von Kindern mit Verdacht auf Retinoblastom sollte immer multidisziplinär erfolgen: Im Mittelpunkt der Diagnostik steht immer die klinische Abklärung, wodurch die Diagnose bzw. der Ausschluss eines Retinoblastoms in den meisten Fällen möglich ist. Klassische klinische Symptome bzw. Zeichen des Retinoblastoms sind Leukokorie (weißliches Aufleuchten der Pupille auf Fotos mit Blitzlicht), Schielen, eine Sehstörung sowie bei weiter fortgeschrittenen Erkrankungen eine Rötung oder Schwellung des Auges und Augenschmerzen, häufig bei erhöhtem Augeninnendruck. Die augenärztliche Erstuntersuchung findet immer in Kurznarkose mit medikamentös erweiterten Pupillen statt, um die Netzhaut sicher komplett beurteilen zu können und auch peripher gelegene Tumoren detektieren zu können. Im Regelfall wird die Untersuchung mit einer Druckmessung, Ultraschalluntersuchung und Fundusbildgebung und ggf. einer OCT („optical coherence tomography“), eines auf der Technik der Weißlicht-Interferometrie beruhenden Verfahrens zur optischen Darstellung von Gewebeschichten, komplettiert. Neben der augenärztlichen Untersuchung spielt die MRT der Augen und des Kopfes eine zentrale Rolle in der initialen Abklärung zur Bestätigung des Retinoblastoms, des Ausschlusses anderer Differenzialdiagnosen und zur Beurteilung der lokalen und zentralen Tumorausbreitung.

## Klassifikation

Für die Wahl der bestmöglichen Therapie ist eine exakte Bestimmung der Ausdehnung der Erkrankung zum Diagnosezeitpunkt essenziell. Aufgrund der Entwicklung der Therapiemöglichkeiten wurde die Reese-Ellsworth-Klassifikation zunehmend durch die ICRB (International Classification of Retinoblastoma) ersetzt (meist verwendete Version: Philadelphia), da sie bei Einsatz einer Chemotherapie sowie zusätzlicher Lokaltherapie eine bessere Vorhersage eines möglichen Augenerhalts erlaubt [6]. Man unterscheidet in dieser Klassifikation 5 Gruppen:

- *Gruppe A und B:* lokal auf die Retina beschränkte Tumoren (Differenzierung nach Tumordicke/Abstand von der Fovea/N. opticus),
- *Gruppe C:* lokale Ausbreitung,
- *Gruppe D:* diffuse Ausbreitung (subretinal und/oder vitreal),
- *Gruppe E:* extensive, darüber hinausgehende Ausbreitung [7].

Eine häufig ergänzend angewandte Klassifikation ist die TNM-Klassifikation (The American Joint Commitee of Cancer Staging System; [8]). Da Lymphknoten- und Fernmetastasen beim Retinoblastom sehr selten sind, kommt insbesondere dem T‑Stadium eine wichtige Rolle zu, welches entweder klinisch (cT1‑4, mit dem Zusatz H für die Heredität (Hx:*RB1*-Gen-Mutation unklar/unbekannt, H0: normale *RB1*-Allele im Blut, H1: bi-/trilaterales Retinoblastom, positive Familienanamnese oder nachgewiesene *RB1*-Mutation)) oder histopathologisch (pT1-4) bestimmt werden kann.

## Therapie

Alle Kinder mit der Verdachtsdiagnose Retinoblastom sollten in spezialisierten Zentren von einem multidisziplinären Team (Augenärzte, Kinderonkologen, Radiologen, Strahlentherapeuten, Pathologen und Genetikern) behandelt werden, um ein bestmögliches Outcome zu ermöglichen. Das primäre Ziel der Behandlung ist immer eine Minimierung der Mortalität und Morbidität.

### Lokaltherapie

Lokale Therapieoptionen wie Kryotherapie, Laserkoagulation oder Brachytherapie können, je nach Tumorlage, für isolierte Tumorherde der Gruppe A oder B eingesetzt werden. Gleichfalls können diese lokalen Therapieformen im Sinne einer Chemoreduktion nach zuvor verabreichter systemischer oder lokaler Chemotherapie zur Verkleinerung des zu behandelnden Tumors als konsolidierende Therapieoptionen eingesetzt werden. Häufig wird die systemische Chemotherapie mit einer Thermochemotherapie kombiniert. Hierbei werden die Tumoren mittels eines Lasers einer speziellen Wellenlänge erhitzt, um eine bessere Penetration des zuvor verabreichten Chemotherapeutikums in den Tumor zu ermöglichen [9].

### Chemotherapie

**Figure 1:**
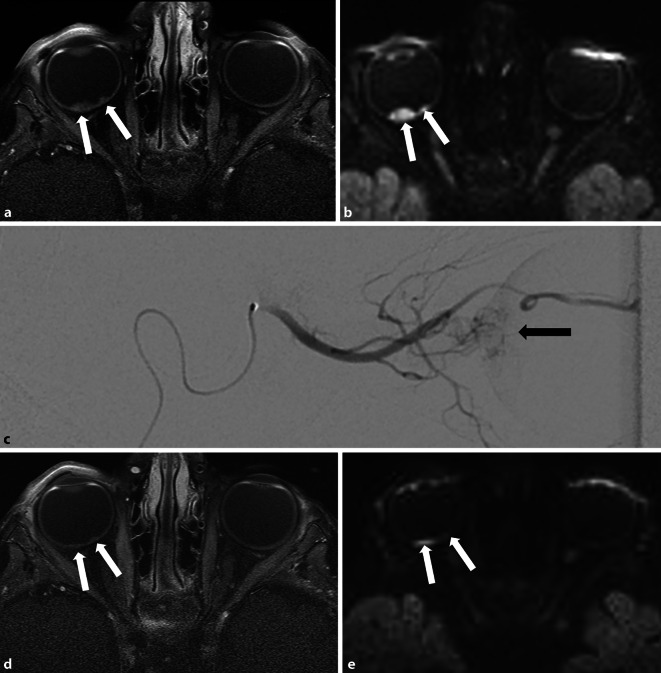

Die Chemotherapie ist Bestandteil der meisten Therapieansätze. Zunehmend werden lokale Applikationsformen eingesetzt, wie die direkte arterielle Applikation in die A. ophthalmica (intraarterielle Chemotherapie, IAC; Abb. 1). Der Vorteil ist, dass das Chemotherapeutikum in einer höheren Konzentration in das Auge appliziert werden kann, die systemischen Nebenwirkungen aber geringer sind [10]. Die IAC kann als primäre Therapieoption bei Augen der Gruppe B bis D eingesetzt werden oder sekundär bei Tumorrezidiven und subretinaler Tumorzellaussaat. Andere Möglichkeiten sind die intravitreale – insbesondere bei Vorliegen einer Glaskörperaussaat – oder periokuläre Chemotherapie.

### Strahlentherapie

Die Strahlentherapie wird aufgrund des höheren Risikos für ein Zweitmalignom zunehmend seltener angewendet. Sie spielt jedoch weiterhin bei Patienten mit histopathologischen Risikofaktoren nach erfolgter Enukleation eine Rolle zur Vermeidung eines Lokalrezidivs, bei intrakraniellen Tumormanifestationen und Metastasen. Eine neue Form der Strahlentherapie ist die Protonentherapie, die aktuell auch bei Retinoblastomen eingesetzt werden kann.

### Enukleation

Die Enukleation ist trotz der Weiterentwicklung der Therapie weiterhin die primäre Therapieoption für unilateral erkrankte Augen der Gruppe E und einige Fälle der Gruppe D. Sie ermöglicht die nachfolgende histopathologische Evaluation der Tumorausdehnung mit Identifikation histologischer metastatischer Risikofaktoren und damit eine bestmögliche Planung der Therapie zur Vermeidung eines Lokalrezidivs bzw. einer Metastasierung, welche die Prognose des Überlebens deutlich verschlechtern würden (Mortalität 75 % bei orbitalen Rezidiven [11]). Nach der Enukleation erfolgt die Versorgung mit orbitalen Implantaten, um die psychosoziale Beeinträchtigung der Patienten sowie die Gefahr einer Gesichtsdeformität so gering wie möglich zu halten.

## Bildgebung

Trotz der nachweisbaren charakteristischen Verkalkungen sollte eine Computertomographie (CT) bei Kindern mit Retinoblastom zur Diagnose nicht durchgeführt werden, da zum einen die Diagnose in der augenärztlichen Untersuchung in Kombination mit der MRT gestellt wird und zum anderen die CT in der Evaluation der lokalen Tumorausbreitung der MRT-Untersuchung deutlich unterlegen ist [12]. Zudem sollte gerade bei Kindern mit einem Tumorprädispositionssyndrom jede unnötige Strahlenbelastung (aufgrund der Gefahr der Tumorinduktion) vermieden werden. Sonographische Untersuchungen können ergänzend in der Diagnostik eingesetzt werden.

Zahlreiche Studien haben gezeigt, dass die hochaufgelöste MRT-Untersuchung der Orbita mit ergänzenden Kopfsequenzen die Bildgebungsmodalität der Wahl bei Kindern mit Retinoblastom ist [12, 13]. Die Aufgaben der Bildgebung sind zum Diagnosezeitpunkt die Bestätigung der Diagnose und das Staging, im weiteren Verlauf der Ausschluss von Rezidiven und Sekundärmalignomen.

### Grundlagen der MRT-Bildgebung des Retinoblastoms

**Figure 2:**
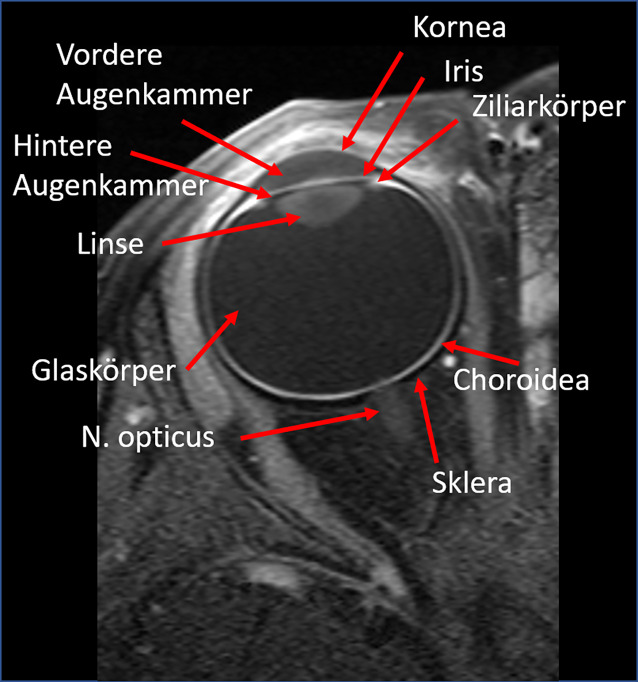

Entsprechend den Guidelines zur Bildgebung des Retinoblastoms der European Retinoblastoma Imaging Collaboration (ERIC) sollten MRT-Untersuchungen bei Kindern mit Retinoblastom immer als hochaufgelöste Orbitauntersuchungen erfolgen [14]. Dies ermöglicht die beste Darstellung des Bulbus oculi. Es sollte insbesondere auf eine gute Beurteilbarkeit der Papille und des retrobulbären N. opticus geachtet werden sowie auf die Differenzierbarkeit der gut perfundierten und somit in den Kontrastmittel(KM)-unterstützten T1-gewichteten Sequenzen gut als hyperintense okuläre Wandschicht abgrenzbaren Choroidea und der außen angrenzenden hypointensen Sklera (Abb. 2). Im Interesse optimaler Bildqualität wird die Untersuchung in Narkose empfohlen.

Das empfohlene MRT-Protokoll findet sich in den Guidelines der ERIC-Gruppe. Die Untersuchung sollte an 1,5-Tesla-Systemen mit Orbita‑/Oberflächenspulen oder an 3‑Tesla-Systemen mit einer Multikanalkopfspule erfolgen. Sollte eine solche Untersuchung aufgrund von technischen Limitationen nicht möglich sein, ist eine Verlegung des Patienten in das nächste Zentrum zur MRT-Diagnostik zu erwägen. Angelehnt an die Guidelines, enthält das minimal notwendige MRT-Protokoll der Orbita folgende Sequenzen:

- axiale T2-Sequenzen: (F)SE (Fast Spin-Echo) mit TE (Echozeit) ≥ 120 ms, optional zusätzlich CISS (Constructive Interference in Steady State), FIESTA (Fast Imaging Employing Steady-state Acquisition) oder DRIVE ≤ 1 mm Schichtdicke (SD),
- axiale native T1-Sequenz sowie nach KM-Gabe axiale und parasagittale (längs zum Verlauf des N. opticus) T1-Sequenzen ohne Fettsättigung.

Wichtig ist für die orbitalen Sequenzen, dass die Schichtdicke maximal 2 mm ist, die Pixelgröße ≤ 0,5 × 0,5 mm beträgt und dass die Sequenzen parallel zum distalen Ende (distale 1 cm) des N. opticus ausgerichtet werden. Die CISS/FIESTA/DRIVE hat den Vorteil der 3‑D-Rekonstruktion, außerdem werden kleine Befunde wie weitere Tumorherde oder eine vitreale/subretinale Tumoraussaat sensitiver erfasst. Nach eigener Erfahrung sind außerdem axiale T1-gewichtete Sequenzen mit Fettsättigung nach KM hilfreich (zur Abklärung bulbusüberschreitenden Wachstums, insbesondere bei orbitaler Zellulitis) sowie Diffusionssequenzen zur Beurteilung der Tumorvitalität.

Für die zusätzliche Untersuchung des Kopfes werden als Minimalprogramm eine axiale T2-Sequenz (SD ≤ 4 mm) und eine T1-Sequenz nach KM (2-D-Spin-Echo [SE] mit SD ≤ 3 mm oder 3‑D-Gradientenecho [GRE] mit SD ≤ 1 mm) empfohlen. Optionale Zusatzsequenzen sind koronare/sagittale T2-Sequenzen, vor allem bei Auffälligkeiten der Glandula pinealis.

### Diagnose eines Retinoblastoms

**Figure 3:**
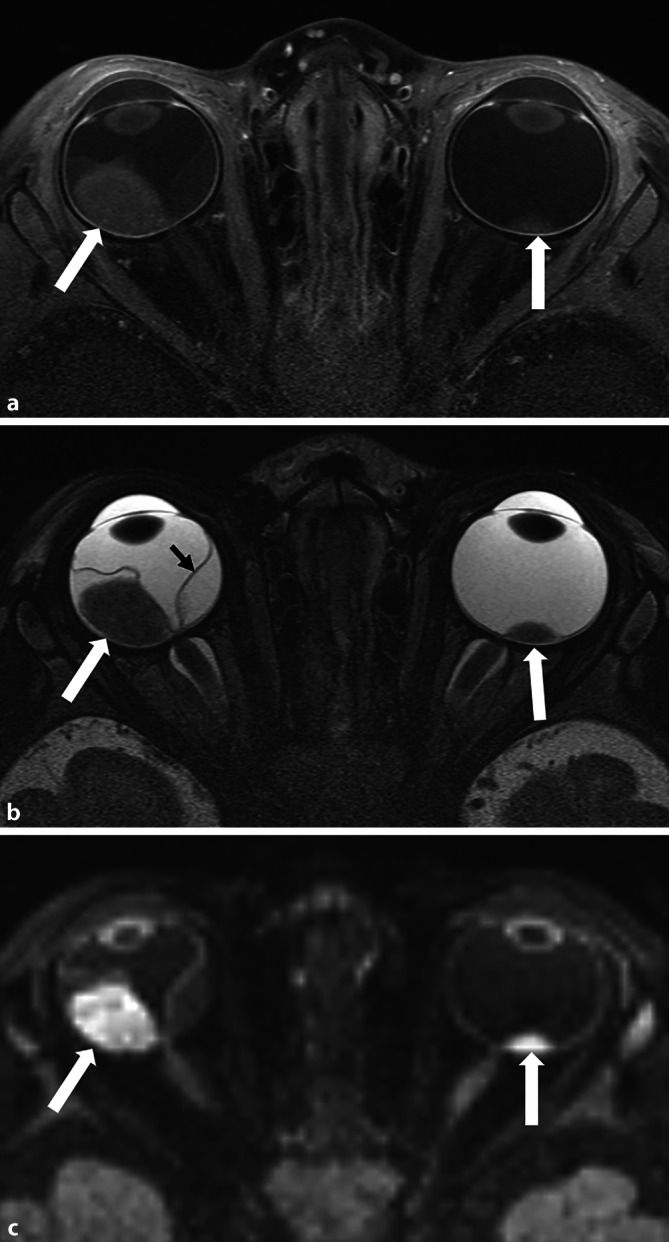

Das Retinoblastom stellt sich in nativen T1-gewichteten Sequenzen normalerweise hyper-, in T2-gewichteten Sequenzen hypointens zum Glaskörper dar mit meist inhomogener KM-Anreicherung sowie Diffusionsrestriktion (Abb. 3). Es können 2 Wachstumsarten unterschieden werden: endophytisch – nach innen gerichtet (häufiger vitreale Tumoraussaat) und exophytisch – nach außen, in Richtung Sklera gerichtet (häufiger Retinaablösung mit subretinaler Flüssigkeit/Seeding); häufig finden sich Mischtypen. Eine vermehrte Anreicherung des vorderen Augenabschnitts kann Ausdruck einer Beteiligung dieses Bereichs mit Neovaskularisation der Iris sein, eine Abflachung der vorderen Augenkammer und/oder eine orbitale Zellulitis ein Zeichen einer intraokulären Druckerhöhung. Wichtig bei der Befundung ist eine Evaluierung der Lateralität, der Tumoranzahl und -lokalisation (insbesondere auch eine mögliche Involvierung der Papille) und der Tumorausbreitung.

### Differenzialdiagnose

Die wichtigsten Differenzialdiagnosen des Retinoblastoms sind die persistierende fetale Vaskularisation (PFV), der M. Coats (eine seltene angeborene Augenerkrankung der Netzhautgefäße) und andere intraokulare Tumorerkrankungen, wie beispielsweise das Astrozytom. Bei der Differenzierung spielt die MRT eine große Rolle, da bei ausgedehnter Begleitablatio und fortgeschrittener Erkrankung in einigen Fällen eine Diagnose nur über die augenärztliche Untersuchung nicht mit Sicherheit gestellt werden kann. Während eine Vergrößerung des betroffenen Auges, eine V‑förmige Retinaablösung, eine vitreale Tumoraussaat und das Vorliegen KM-anreichernder solider Weichteilanteile für ein Retinoblastom sprechen, sind eine Verkleinerung des Auges, eine Y‑förmige Retinaablösung, intraretinale Makrozysten, ein Missmatch zwischen KM-Anreicherung und soliden Anteilen bzw. ein Fehlen der KM-Anreicherung solider Anteile eher Hinweise auf ein *Pseudoretinoblastom*. Deformitäten des Ziliarkörpers und/oder der Linse, eine Optikusatrophie und ein zentraler Strang (in dem während der Entwicklung physiologische fetale Gefäße verlaufen) sprechen für das Vorliegen einer PFV, insbesondere bei Kombination der vorliegenden Merkmale. Das Vorhandensein von KM-anreichernden, subfovealen Knötchen kann ein Hinweis auf einen M. Coats sein [15].

### Evaluation lokaler Tumorausdehnung

Da die Prognose und Therapiemöglichkeiten okulärer Tumoren ohne und mit fokaler lokaler Invasion gut sind, ist die Aufgabe der MRT-Untersuchung vor allem die Detektion einer signifikanten lokalen Invasion, eines extraokulären Tumorwachstums sowie der Ausschluss einer trilateralen Erkrankung bzw. Metastasierung. Unter einer signifikanten lokalen Invasion versteht man das Vorliegen einer massiven Choroideainfiltration, einer retrolaminären Optikusinfiltration oder einer Sklerainvasion.

### Choroideainfiltration

**Figure 4:**
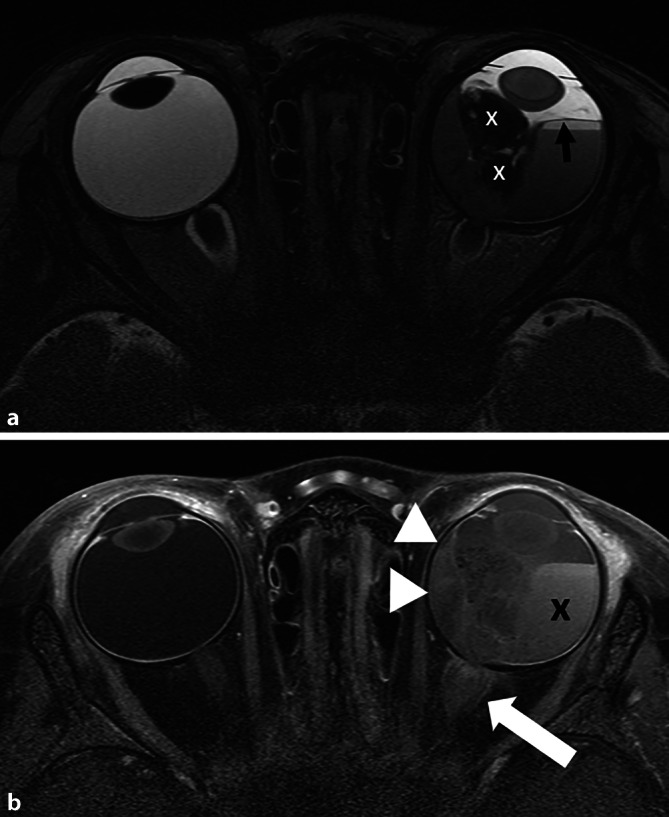

Bei der Choroidea unterscheidet man eine fokale (C1) von einer massiven (C2: Ausdehnung ≥ 3 mm und/oder Erreichen der Sklera) Infiltration, wobei nur letztere ein signifikanter metastatischer Risikofaktor ist [8]. In der MRT kann sich die Choroideainfiltration auf 2 Arten darstellen:

1. als fehlende Abgrenzbarkeit der normalen Choroidea (häufiger; Abb. 4) oder
2. als deren fokale Verdickung und Mehranreicherung (seltener; [16]).

Gerade für die zuverlässige Beurteilung einer Choroideainfiltration ist eine hochaufgelöste MRT-Bildgebung wichtig [13].

### Sklerainfiltration

Insbesondere bei vorliegender Choroideainfiltration ist die detaillierte Inspektion der Sklera wichtig, da sie die Barriere zum extraokulären Fettgewebe darstellt, dessen Invasion die Prognose deutlich verschlechtern würde. Die Sklera kann, wie auch die Choroidea, am besten auf T1-Sequenzen nach KM-Gabe beurteilt werden, wo sie sich als hypointense Struktur darstellt. Man unterscheidet eine S1-Infiltration (Infiltration der inneren zwei Drittel) von einer S2-Infiltration (Infiltration über vollständige Breite, skleraüberschreitendes Wachstum; [8]).

### Optikusinfiltration

Die zweite Ausbreitungsmöglichkeit des Tumors nach extrabulbär ist eine Infiltration des N. opticus (N1: prälaminär bis in die Lamina cribrosa, N2: postlaminär, Abb. 4, histologisch noch N3: Tumor am Resektionsrand des N. opticus; [8]). Die postlaminäre Optikusinfiltration ist ein relevanter metastatischer Risikofaktor und zeigt sich als vermehrte Anreicherung im N. opticus mit teils zusätzlicher Auftreibung. Diese kann sich teils sehr dezent darstellen und somit auch der Darstellbarkeit in der MRT entziehen, wobei die Detektionsrate bei Anwendung hochaufgelöster MRT-Technik der Standardtechnik überlegen zu sein scheint [12, 13].

### Trilaterales Retinoblastom

**Figure 5:**
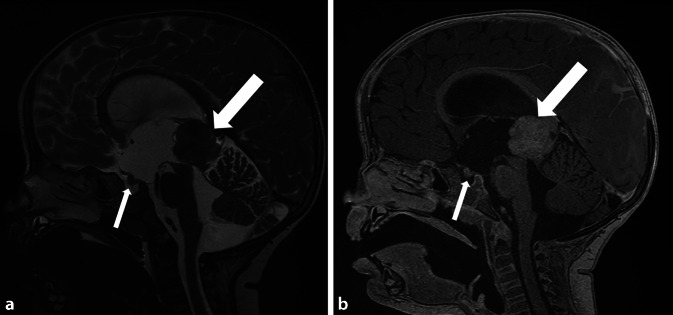

Bei Patienten mit hereditärer Retinoblastomerkrankung ist das Risiko für das Vorliegen einer trilateralen Erkrankung, d. h. eines weiteren Tumorherds intrakraniell erhöht. Die häufigsten Lokalisationsorte hierfür sind die Glandula pinealis (Pinealoblastom; Abb. 5) und suprasellär. Im Fall eines Pinealoblastoms unterscheidet man eine solide von einer zystischen Manifestationsform. Die Verwendung von Normwerten für die Größe der Glandula pinealis kann zur Differenzierung hilfreich sein, ebenso wie das Vorliegen KM-affiner solider Tumoranteile und ein Progress über die Zeit. Eine Hilfestellung für das klinische Management/Follow-up von Retinoblastompatienten mit unklarem Befund der Glandula pinealis bietet das Flowchart der ERIC-Gruppe [17, 18].

Neben der Evaluation des Vorliegens einer trilateralen Erkrankung ist der Ausschluss einer Meningeosis carcinomatosa und/oder assoziierter Malformationen, etwa bei 13q-Deletionssyndrom, relevant. Zusätzlich sollte stets der gesamte erfasste Untersuchungsbereich auf Metastasen abgesucht werden.

### Bildgebung im Verlauf

**Figure 6:**
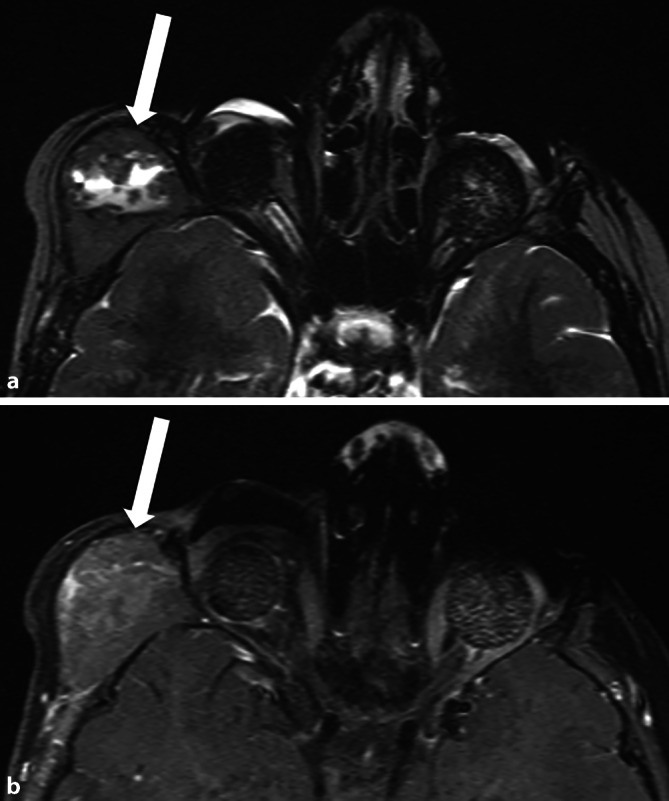

Die MRT-Bildgebung hat bei Kindern mit Retinoblastomen auch im Verlauf einen hohen Stellenwert, vor allem zur Therapieevaluation (Abb. 1), dem Rezidivausschluss oder von Komplikationen nach Implantateinlage. Die ERIC-Gruppe empfiehlt aufgrund der schlechten Prognose orbitaler Rezidive und deren Auftreten im Regelfall innerhalb von 2 Jahren nach Enukleation regelmäßige MRT-Verlaufskontrollen 3 Monate nach Enukleation und im Anschluss alle 6 Monate (bei unauffälligem Befund) bis 2 Jahre nach Enukleation oder sofort bei klinischem Verdacht auf ein Rezidiv [19]. Weitere wichtige Indikationen zur MRT-Bildgebung von Kindern mit Retinoblastom sind Verlaufskontrollen des intrakraniellen Befunds bei hereditärem Retinoblastom. Ob ein systematisches MRT-Ganzkörper-Screening auf Sekundärmalignome bei Vorliegen eines Tumorprädispositionssyndroms erfolgen sollte, wird aktuell noch kontrovers diskutiert [20]; bei klinischen Symptomen sollte jedoch stets eine gezielte MRT-Abklärung der Region erfolgen, um Zweitmalignome nicht zu verpassen (Abb. 6).

## Ausblick

Insgesamt geht der Trend der Therapie in den letzten Jahren aufgrund der hohen Überlebensraten immer mehr zur augenerhaltenden Therapie, da diese für den Patienten funktionell und in Hinsicht auf die Lebensqualität ein relevanter Gewinn ist. In diesen Fällen steht dann allerdings kein Tumormaterial zur histologischen Analyse und Evaluation der Tumorausdehnung zur Verfügung, da eine Biopsie kontraindiziert ist. Der Bildgebung kommt somit, insbesondere bei diesen Patienten, eine steigende Bedeutung zu. Aktuelle Forschungsprojekte im Bereich Radiogenomics bemühen sich außerdem um eine zunehmende Phänotyp-Genotyp-Korrelation mittels MRT, um diese Lücke zu füllen und zu versuchen, aggressive genetische Subtypen frühzeitig zu erfassen [21]. Des Weiteren besteht die Hoffnung, auch durch den Einsatz künstlicher Intelligenz (KI) im Rahmen von Forschungsprojekten und nachfolgend auch in der klinischen Routine, eine Verbesserung der Bildqualität, der Befunddetektion und -interpretation sowie neue Erkenntnisse für die Phänotyp-Genotyp-Korrelation zu erreichen [22, 23].

## Fazit für die Praxis

- Die hochaufgelöste Magnetresonanztomographie (MRT) spielt bei Kindern mit Retinoblastom eine Schlüsselrolle in Diagnostik, Staging, Therapieplanung und -evaluation.
- Eine computertomographische Untersuchung ist nicht indiziert.
- Die wichtigsten zu beurteilenden Punkte sind okulär die Lateralität, die Anzahl/Lokalisation der Tumoren, die lokale Ausdehnung (postlaminäre Optikusinfiltration, massive Choroideainfiltration, Sklerainfiltration) und intrakraniell das Vorliegen/der Ausschluss eines trilateralen Retinoblastoms bzw. (leptomeningealer) Metastasen.
- Alle Kinder mit der Verdachtsdiagnose Retinoblastom sollten in spezialisierte Zentren zur Diagnoseabklärung und Therapieplanung überwiesen werden.
- Bei der Therapieplanung sowie der Betreuung der Patienten in ihrem weiteren Leben ist bei Kindern mit Retinoblastom das Vorhandensein eines Tumorprädispositionssyndroms abzuklären, um für die Patienten das bestmögliche funktionelle Outcome und die höchstmögliche Lebensqualität zu erreichen.
